# Is Sickle Cell Anemia a Neglected Tropical Disease?

**DOI:** 10.1371/journal.pntd.0002120

**Published:** 2013-05-30

**Authors:** Russell E. Ware

**Affiliations:** 1 Texas Children's Center for Global Health, Texas Children's Hospital, Houston, Texas, United States of America; 2 Department of Pediatrics, Baylor College of Medicine, Houston, Texas, United States of America

Governments and philanthropic organizations collectively pour hundreds of millions of dollars annually into the prevention and treatment of a select group of life-threatening infectious diseases that includes malaria, HIV/AIDS, and tuberculosis. The merits of these high-profile programs are indisputable, yet emerging interest in traditionally neglected tropical diseases (NTDs) suggests that the lights of global awareness and medical treatment are now shining more brightly on lesser-known infections, which remain scourges for more than a billion persons worldwide [1]. More recently, noncommunicable diseases also have garnered attention and funding as causes of serious morbidity and mortality, including diabetes, cardiovascular disease, and cancer [2]. Standing awkwardly alone between these traditionally communicable and noncommunicable diseases, however, is sickle cell anemia (SCA). As a congenital but noninfectious disease with high childhood mortality, SCA is a serious health condition that represents a silent killer of children with enormous global burden. Only with improved recognition and a concerted worldwide effort can we begin to address this disparity and offer life-saving interventions to millions of affected children.

Should SCA be considered an NTD? From the viewpoint of this hematologist, an NTD can be operationally defined as any serious medical affliction with the following characteristics: (1) worldwide distribution, typically affecting millions; (2) highest burden among the most impoverished and disadvantaged populations; (3) serious morbidity, diminished quality of life, and even mortality; (4) comorbidity for other life-threatening diseases; (5) relatively simple diagnostic testing; and (6) inexpensive treatment options. Although NTDs are usually infectious and communicable, perhaps those should not be strict requirements. Indeed, based on these criteria, SCA is long overdue for membership in this elite but tragic club of global medical maladies. Perhaps ironically, such a designation of SCA may allow this currently “invisible” lethal disease to become more noticeable as a “neglected” disease.

## How Did Sickle Cell Anemia Arise?

SCA results from the homozygous inheritance of a single DNA mutation within the beta globin gene, leading to a glutamic acid to valine substitution within the hemoglobin tetramer, changing normal hemoglobin (HbA) into abnormal sickle hemoglobin (HbS). In deoxygenating conditions such as the venous circulation, HbS rapidly polymerizes within the erythrocytes, leading to intracellular tactoids that deform the red blood cells into the characteristic curved or sickled shape. Persons with SCA suffer a wide variety of serious disease complications: increased susceptibility to bloodstream infections, especially early fatal pneumococcal sepsis; chronic hemolytic anemia that features both intravascular and extravascular erythrocyte destruction; recurrent periodic acute vaso-occlusive events, including pain and acute chest syndrome; and chronic damage affecting almost every organ system.

**Figure 1:**
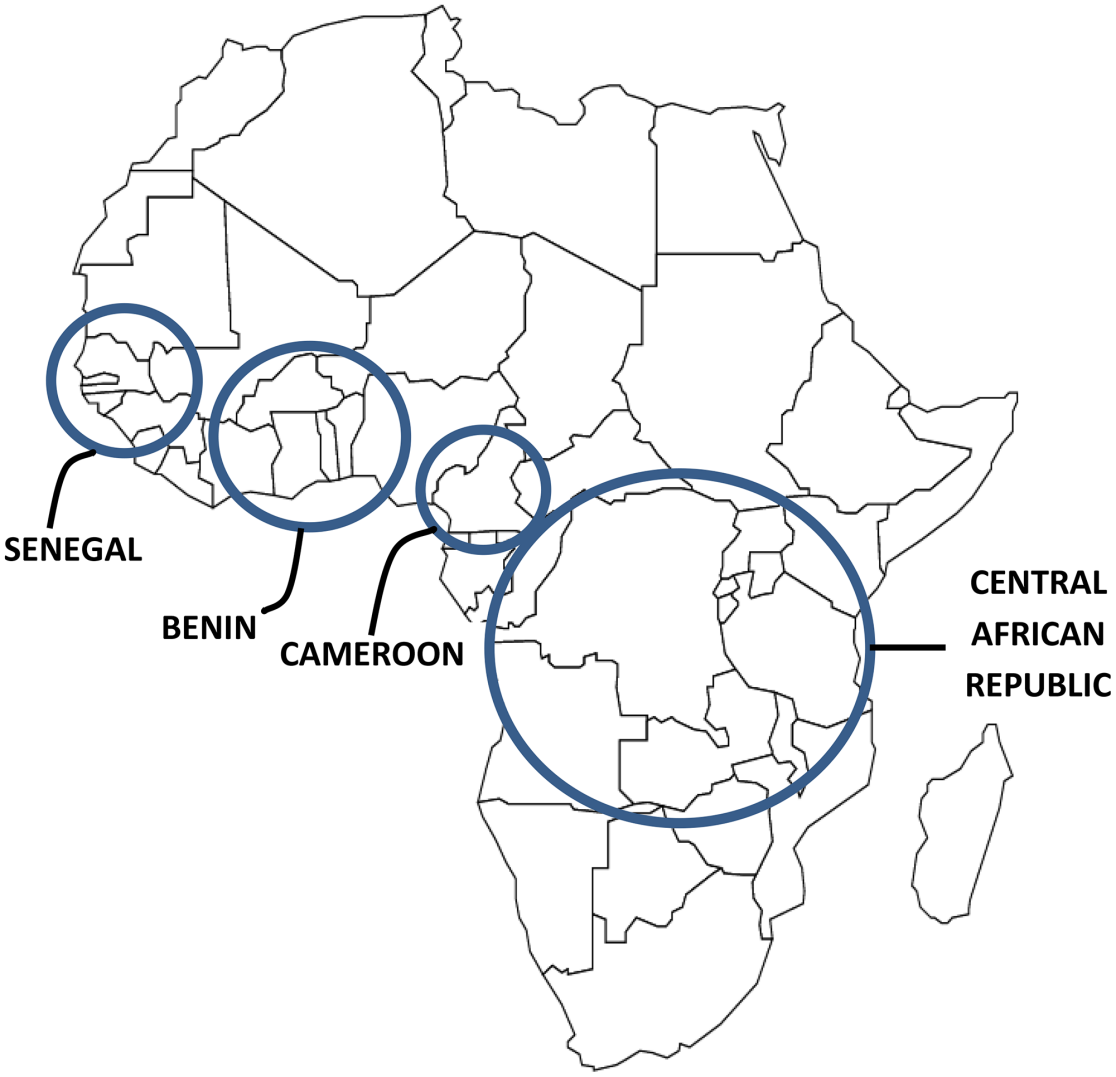
Multiple origins of the beta globin Glu6Val (β^s^, sickle) mutation within the African continent. Malaria selection pressure led to the emergence of the sickle mutation at four distinct locations in sub-Saharan Africa, which are distinguishable by flanking DNA polymorphisms that define beta globin SCA haplotypes from Senegal (SEN), Benin (BEN), Cameroon (CAM), and Central African Republic (CAR). An additional sickle mutation occurred in the Arabian-Indian region, not shown.

As illustrated in Figure 1, the sickle mutation arose independently in several regions of the world, including at least four distinct locations within sub-Saharan Africa as well as the Arab peninsula and Indian subcontinent. This multicentric origin is explained by genetic selection pressure: the allele frequency of the heterozygous sickle mutation (HbAS, or sickle cell trait) closely matches the regions of highest malaria endemicity [3], supporting the hypothesis that HbAS confers protection against severe malaria with *P. falciparum*. The relative survival advantage of persons with HbAS, which results from a series of clever biochemical and genomic mechanisms that reduce malarial pathophysiology (reviewed in [4]), helps to explain both its geographic origins and its ongoing selection pressure in malaria-endemic regions of the world. But the homozygous sickle mutation (HbSS, the most common and severe form of SCA) is a risk factor for death from malaria [5], as well as a potent comorbid factor for death from bacterial infections, particularly invasive pneumococcal disease [6]. The sickle mutation is thus a balanced genetic polymorphism, such that a single inherited allele promotes an individual's survival and increases the chance of genetic propagation, while a double copy negatively affects survival and transmission. Recent data estimates suggest over 5.5 million HbAS births and 313,000 HbSS births annually, with 75% of these occurring in sub-Saharan Africa [7].

## The Challenge

In 2006, the World Health Organization issued a report that specifically addressed SCA as a prevalent medical condition with clinical severity, contributing to the under-5 deaths on the African continent. This document identified an “urgent need to develop models of care appropriate to the management of the disease in sub-Saharan Africa,” and recommended gradual introduction of services where feasible, emphasizing community education and partnership [8]. The need for research and surveillance was also highlighted. Subsequently, the global burden has been quantified, with SCA accounting for 6.4% of the under-5 mortality across all of Africa [9]. However, in certain countries with higher sickle allele frequencies and lower childhood mortality rates, such as Uganda, it is likely that SCA contributes to up to 15% of the under-5 mortality rate. Unfortunately, the vast majority of these cases are undiagnosed, and instead the causes of childhood mortality are attributed only to pneumonia or malaria, rather than the more accurate underlying SCA.

What can be done? In the United States, universal newborn screening programs accurately identify all infants soon after birth, which enables early education and treatment programs to commence before disease complications occur. For example, newborn diagnosis allows early intervention with penicillin prophylaxis and pneumococcal immunization, as well as education regarding infection and splenic sequestration and transcranial Doppler screening for primary stroke risk. In developed countries, these approaches now allow 95%–99% of children with SCA to survive into adulthood [10], [11], making this a chronic hematological disorder warranting long-term therapeutic and management strategies. In contrast, survival of children with SCA born in developing countries is dismal; an estimated 50%–90% of affected children will die before age 5 years, due either to complications of SCA itself or more commonly from pneumococcal disease, malaria, or diarrheal disease [12].

Perhaps simple interventions that work in Western countries can also work in developing countries in sub-Saharan Africa. Pilot data from Benin [13], Kenya [6], and Democratic Republic of Congo [14] suggest that neonatal screening in sub-Saharan countries is feasible and may lead to improved clinical outcomes. A well-developed newborn screening program now exists in parts of Ghana [15], and a recently established South-South partnership between Ghana and Brazil should lead to the first national screening program in Africa. In 2011, a unique private-public partnership was developed among the Republic of Angola, Chevron Corporation, and Baylor College of Medicine. A pilot newborn screening program was initiated in the capital city of Luanda, Angola using dried blood spots collected primarily from two large maternity hospitals. In the first year, over 17,000 newborns were screened for hemoglobin disorders; an enormous burden was identified, with over 21% HbAS and 1.5% HbSS among this newborn cohort [16]. Families of affected infants were contacted and relatively inexpensive treatments were provided, including penicillin prophylaxis, pneumococcal immunizations, malaria bed nets, and education about the importance of seeking medical attention for fever. Retention and survival of affected infants has exceeded 95% in the first year of life [16]. These preliminary but encouraging results suggest that simple and life-saving interventions can be successfully provided in sub-Saharan Africa for infants and children with SCA.

## A Call to Action

In 2008, the United Nations recognized SCA as a global health priority, and World Sickle Cell Awareness Day (June 19) now commemorates this recognition. In 2010, WHO issued a supplemental report on SCA that provided specific targets and goals for sub-Saharan countries, related to a national strategy for comprehensive care and treatment of this disorder [17]. By 2015, 25% of countries should have a plan, and by 2020, 50% of countries should have a plan to reduce under-5 mortality by 30%. Such bold targets will not be met easily, however. Active North-South and South-South partnerships that prioritize research will be required to help sub-Saharan countries develop robust sickle cell strategies that can provide diagnosis, management, and treatment of SCA [18].

Despite its origins in Africa and Arab-India, SCA is now recognized as a truly worldwide health problem. Tens of thousands of SCA births occur in the Middle East and across the Americas each year. Recent immigration patterns have now led to sizable sickle cell populations emerging in previously unaffected areas of the world, including Ireland, Scandinavia, Australia, and South Africa. Active research partnerships can begin with networking, as supported by the new Global Sickle Cell Disease Network [19]. More research is needed, beginning with better baseline data on the global burden of SCA, especially regarding its epidemiology and contributions to under-5 mortality rates and disease morbidity. Official childhood mortality reports should include SCA as a specific and measureable cause of death, similar to the data currently reported for malaria, HIV/AIDS, and measles [20]. Disease morbidity summaries should include SCA as a specific cause, since recent data on the global burden of disease [21] indicate that disability-adjusted life years (DALYs) from sickle cell disease are similar to those from cervical cancer and greater than those from chronic kidney disease due to diabetes mellitus or hypertension, all of which have a much higher public awareness.

Interventions for SCA must also include treatment of the underlying disease. The introduction of newborn screening and early life-saving interventions into sub-Saharan Africa will result in a large cohort of surviving children with SCA, which will only add to the healthcare burden of low-income countries [22]. Hydroxyurea is an attractive therapeutic option for the treatment of SCA, and has been shown to be both safe and efficacious for many laboratory and clinical manifestations of SCA in affected infants and children [23], [24]. However, hydroxyurea has not been tested in developing countries where comorbidities including malaria and nutritional deficiencies may affect the toxicity profile. Prospective studies such as the proposed Realizing Effectiveness Across Continents with Hydroxyurea (REACH) trial will gather critical data regarding the feasibility, safety, and efficacy of hydroxyurea in sub-Saharan Africa.

## Final Considerations

Based on the six criteria proposed initially, there are compelling reasons to consider SCA an NTD. First, SCA has a worldwide distribution in Africa, Asia, and the Americas, with over 300,000 births occurring annually. Second, the highest burden of SCA falls on the poorest and most disadvantaged populations in sub-Saharan Africa and tribal Indian populations. Third, SCA causes serious morbidity and contributes substantially to under-5 mortality rates. Fourth, SCA is an important comorbid factor for other life-threatening diseases, especially malaria and invasive pneumococcal disease. Fifth, rapid and inexpensive testing at birth or in the neonatal period can accurately determine the presence of SCA. Sixth, simple interventions with penicillin prophylaxis, pneumococcal immunizations, and education can be life-saving and lead to improved survival. Most importantly, however, this life-threatening hematological disease has been long overlooked by almost all medical organizations and governments, and is only now receiving modest interest and attention as a substantial global healthcare issue. With better awareness and funding, SCA can change from an invisible killer of children to a neglected one, which will help promote the need for life-saving diagnostic and treatment efforts to reach affected infants and children. We hematologists should learn from NTD specialists and experts to develop strategies and policies that integrate testing and treatment [25], [26], which will allow improved survival and quality of life for disadvantaged children with SCA wherever they live.
